# Gene Expression Profiles for Predicting Metastasis in Breast Cancer: A Cross-Study Comparison of Classification Methods

**DOI:** 10.1100/2012/380495

**Published:** 2012-11-28

**Authors:** Mark Burton, Mads Thomassen, Qihua Tan, Torben A. Kruse

**Affiliations:** ^1^Research Unit of Human Genetics, Institute of Clinical Research, University of Southern Denmark, Sdr. Boulevard 29, 5000 Odense C, Denmark; ^2^Department of Clinical Genetics, Odense University Hospital, Sdr. Boulevard 29, 5000 Odense C, Denmark; ^3^Institute of Public Health, University of Southern Denmark, J. B. Winsløws Vej 9B, 5000 Odense C, Denmark

## Abstract

Machine learning has increasingly been used with microarray gene expression data and for the development of classifiers using a variety of methods. However, method comparisons in cross-study datasets are very scarce. This study compares the performance of seven classification methods and the effect of voting for predicting metastasis outcome in breast cancer patients, in three situations: within the same dataset or across datasets on similar or dissimilar microarray platforms. Combining classification results from seven classifiers into one voting decision performed significantly better during internal validation as well as external validation in similar microarray platforms than the underlying classification methods. When validating between different microarray platforms, random forest, another voting-based method, proved to be the best performing method. We conclude that voting based classifiers provided an advantage with respect to classifying metastasis outcome in breast cancer patients.

## 1. Introduction 

The analysis of high-dimensional gene expression datasets has posed new computational challenges. These datasets have, for example, in breast cancer research, been applied to develop classifiers predicting metastasis outcome, disease recurrence, or breast cancer survival. Some of the classification methods most frequently applied to microarray data are logistic regression [1, 2], support vector machines (SVM) [3–12], neural networks (NNET) [1, 13], random forest (RF) [1, 12], and classifiers based on voting [1]. However, few studies have systematically compared the predictive performance of such methods using microarray gene expression datasets on breast cancer. In their studies, method comparisons have been done within the same datasets by, for example, 10-fold cross-validation, leave-one-out cross-validation, or hold-out procedures [14–18], addressing prediction of relapse within a 5-year period [14, 16, 19], or molecular subtype classification [15]. Furthermore, even fewer studies have compared cross-study validation between classification methods within the field of breast cancer research. Two studies addressed ER-positivity and molecular subtype classification [20, 21], while another tested prediction of relapse within a 5-year period in a small group of 19 independent patients [22]. 

This study compares the performance of seven classification methods belonging to four different categories for predicting metastatic outcome in lymph negative breast cancer patients, which have not been treated with adjuvant systemic therapy. The classification methods used included an ensemble decision tree model (random forest), regression (logistic regression), four support vector machines and a neural network. To address various degrees of variation for such tasks, the comparisons were done either within the same dataset (internal) or between different datasets (external). Within the same dataset model building and classification were performed using 10-fold cross-validation. Across datasets the comparisons were done in two ways. The first is in which the validations are conducted between studies using the same microarray platform (classifiers developed from an Affymetrix dataset and validated on an independent Affymetrix dataset), while the second encompasses validations across studies with different platforms (classifiers developed from an Agilent dataset and validated on an independent Affymetrix dataset). Furthermore, we examined the effect of combining the classification results on each sample by the seven methods into one final classification determined by majority voting, and performances compared by internal and external validation as well.

## 2. Materials and Methods

### 2.1. Datasets Used in This Study

The following eight datasets were used for either defining the gene features and or training purposes in the further study: samples from the studies [23–27] and samples from the Gene Expression Omnibus-(GEO-) series GSE2034 [28], GSE4796 [4], and GSE3494 [6] (Table 1). A subset of 151 node-negative samples from the dataset by van de Vijver (AM) and the entire GSE2034 dataset [28] (abbreviated RO) were used for classifier development in the further study (Table 1). The following datasets were used as independent testing sets: the node-negative samples from GSE7390 [29] (abbreviated TR) and the GSE11121 dataset [30] (abbreviated MA) (Table 1).

### 2.2. Dataset Processing

The eight datasets above were downloaded and directly used for identification of rank-significant genes. Following this identification, the four datasets: AM, RO, TR, and MA were all standardized to have mean zero and standard deviation one. Calculations and classification were all conducted using the R free package. For random forest, logistic regression, support vector machines, and neural network we used the *randomForest*, *glm*, *e1071,* and *nnet* packages, respectively.

### 2.3. Identification of Cross-Study Rank-Significant Features

To determine which genes should be used to build gene expression classifiers, we used the eight publicly available datasets mentioned above, which were used in our two previous studies [34, 35]. This was done by applying the microarray meta-analysis described in [34], upon the individual gene expression values of each individual probe/gene in the eight datasets. This method ranks each individual gene in each dataset according to its signal-to-noise ratio, calculates the gene's mean rank across datasets, and determines if this mean rank is significantly high or low, according to a significance cutoff at FDR ≤ 0.05.

### 2.4. Classifier Building

The features within each training dataset were ranked according to their random forest variable importance measure. For each feature, this value reports the standardized drop in prediction accuracy when the classlabels are permuted [36]. For each feature, this rank was used for model building by subsequently adding one feature at a time in a “top-down” manner. To avoid creating bias, during gene selection and training of the final classifier, and on classification performance, we used ten-times repeated 10-fold cross-validation accuracies as a performance measure, as this metric has previously been shown to give an excellent bias-variance balance [37]. In this study, the models were developed to achieve the best mean sensitivity and specificity thus forcing the overall accuracy to give a balanced sensitivity and specificity. Seven different classification methods were used for model building which included: random forest (RF) [36], logistic regression, SVM with a radial- (R-SVM), a linear (L-SVM), polynomial (P-SVM), or a sigmoid-based kernel (S-SVM) [38], and a neural network with a single hidden layer (NNET). The voting approach is described in detail below. As all classification methods have hyperparameters, we optimized these parameters during model building using a grid-like search of parameter combinations. In random forest, we optimized the number of trees in the forest (*n*tree) from settings of 2000, 3000, 4000, and 5000 trees, and the number of subselected predictors for node splitting (mtry) with settings of: $1,0.5\cdot \sqrt{\left(\text{number}\;\text{of}\;\text{features}\right)}$, $1\cdot \sqrt{\left(\text{number}\;\text{of}\;\text{features}\right)}$, $2\cdot \sqrt{\left(\text{number}\;\text{of}\;\text{features}\right)}$, and total number of features. In all support vector machines, the slack variable penalizing cost parameter (C) was optimized using settings of 0.01, 0.1, 1, and 10, and the *γ*-parameter, controlling the spreading of samples in feature space, with the settings of 0.001, 0.01, 0.1, and 1, and for P-SVM also the polynomial degree using degrees of 2, 3, and 4.

### 2.5. Voting

The voting procedure can be regarded as a metamodel, where a sample is first fed to be predicted by each of the respective classification methods. These predictions are next fed to the final metamodel, combining each of these predictions into a final classification determined by majority voting. 

The voting procedure, at the level of internal dataset prediction, consisted of two steps. In the first step, each sample is classified ten times during 10 × 10 CV, meaning that each sample is given 10 votes for classification within each classification method. To prevent ties, the nine first votes were used for class decision. In the second step the final votes from each of the seven classification methods are combined into one vote, thus creating the cross-classification voting result.

During external validation, every sample is classified once by each of the seven classification methods. The voting classification for each sample is determined by the winning class assigned by the seven classification methods (voters). 

### 2.6. Classification Performance Assessment

We compared the bAcc, defined as the mean of sensitivity and specificity, of classifiers at two levels either internal or external. Internal performance was determined by the 10-times repeated 10-fold cross-validation classification accuracies. External performances obtained through transferring the trained classifier from the training sets to classify each of the independent samples are reported. In external validation, two different situations were examined: (1) between similar (RO on TR or MA) and (2) different microarray platforms (AM on TR or MA), covering Affymetrix-based classifiers validated on an Affymetrix dataset and Agilent-based classifiers validated on another Affymetrix dataset, respectively. 

### 2.7. Endpoint/Outcome Definition

The outcome is defined as metastasis after time of diagnosis. As this study addresses outcome classification, we did not consider the time-to-event component or censoring, due to the fact that survival analysis sometimes can be misleading when considering classification, and because transformation of time-to-event into a binary outcome can blur prediction of the classes [39].

### 2.8. Comparison of External Validation Performance

There is to our knowledge no standard statistics for comparing classifier performance on unbalanced datasets using the balanced accuracy as a performance measure. Therefore, in order to test the significance of the performance difference between the classification methods (defined as a significant difference between correct predictions using method A versus using method B), we used a repeated downsampled binomial test approach consisting of five steps. (1) The classifiers classification results upon the entire test data were initially converted into a balanced test result by downsampling. Downsampling obtains a class-balanced dataset from an imbalanced dataset by removing a subset of randomly selected samples from the majority class, where the number of samples removed equals the difference in sample size between the major and the minor class. In this study the majority class is the nonmetastasis class; (2) the number of samples correctly classified by one classification method but incorrectly by the other classification method and vice versa is counted; (3) the significance of the difference in these counts is determined using a binomial *χ* ^2^-test; (4) the *P*-value of this test is stored. The steps 2 to 4 are repeated 1000 times; (5) from the 1000 tests, the median *P* value is reported as the statistical significance impact between the two compared methods.

## 3. Results

### 3.1. Features and Classifiers/Models

In this study the classifiers were developed to predict metastasis outcome using full follow-up time. To make the classifiers globally applicable and robust, we identified genes being significantly associated with outcomes across eight different studies using different microarray platforms and originating from different populations. These eight datasets are referred to as the “feature definers” (FD) (Table 1). In the further analysis, two of the FD datasets were used as training sets. The first, Rotterdam (RO), is an Affymetrix-based dataset containing 286 samples, and the second, Amsterdam (AM), is a node-negative subset of 151 samples from the entire FD-Amsterdam dataset (Table 1). Two independent datasets, not used for feature selection or classifier development, were used as test sets. These comprise the TRANSBIG (TR) and Mainz (MA) datasets, which are based on the Affymetrix platform and consist of 147 and 200 samples, respectively. 

**Figure 1:**
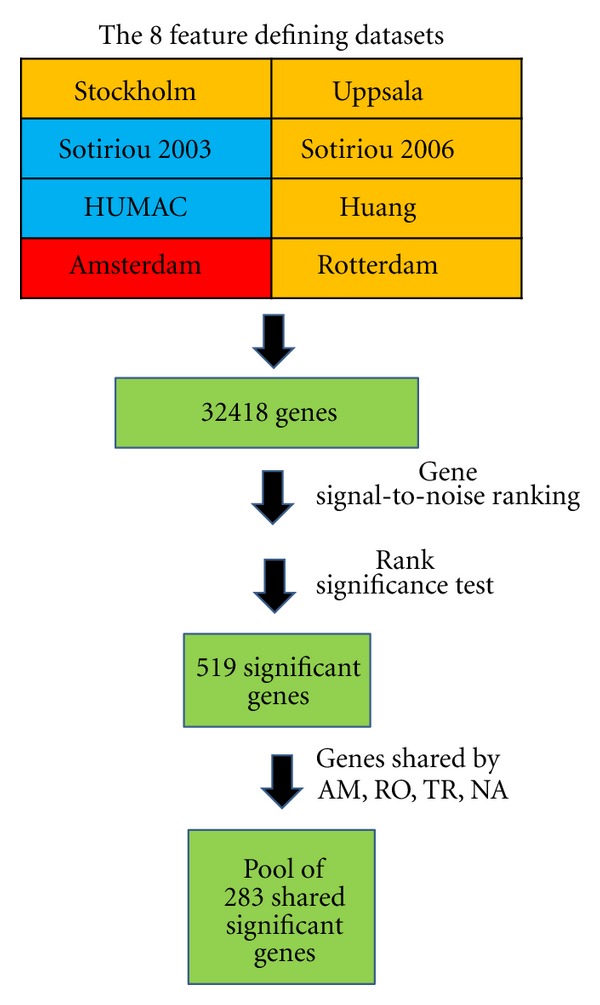
Feature selection. Eight breast cancer gene expression datasets (feature defining datasets), covering 32418 genes, were used to define a list of rank significant genes. Datasets using the Affymetrix platform, spotted oligonucleotides, and the Agilent platform are colored orange, blue, and red, respectively. These genes were first ranked within each of the eight datasets according to their signal-to-noise ratio, and their across dataset mean rank calculated. This mean rank was significance tested as described in Section 2, resulting in a list of 519 rank significant genes. These 519 genes were reduced to a pool of 283 genes shared by the two training sets (AM and RO) and the testing sets (TR and MA), used in the further study.

As a preliminary feature selection step, we identified genes being significantly associated with outcomes across eight different studies using a rank-based method (as described in Section 2). This method led to identification of 519 rank-significant genes. By matching the 519 rank-significant genes and those present in AM, RO, TR, and MA, these genes were reduced to 283 (Figure 1) and were thus used for classifier building. The list of 283 genes is shown in Supplementary Table 1  in Supplementary Material available online at doi:10.1100/2012/380495.

**Figure 2:**
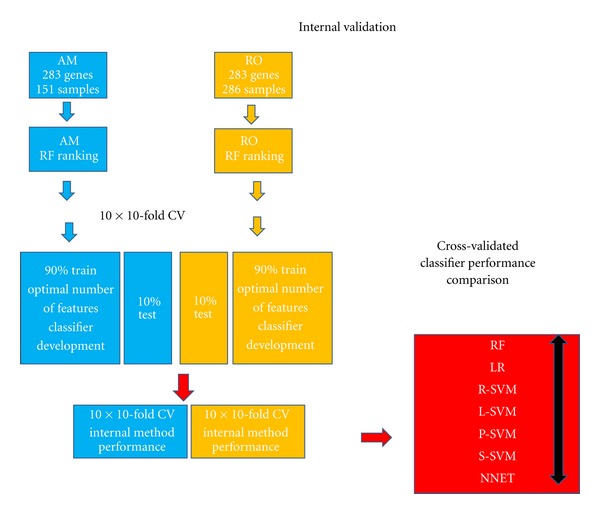
Internal validation procedure. The two datasets, AM (blue) and RO (orange) composed of the 283 rank-significant genes and 151 or 286 samples, respectively, were used for internal performance evaluation. These datasets were first individually used to rank each feature by their random forest variable importance value (RF ranking). These ranks were separately used for selecting the optimal number of features by adding one feature using the same classification method, using a 10-times repeated 10-fold cross-validation procedure. The AM and RO 10-times cross-validation results using the same classification method were combined, and the mean classification performance of each method was compared.

In order to build the models, the 283 features within each training dataset were ranked according to their random forest variable importance measure (Figure 2). For a given feature, this measure reports the standardized drop in prediction accuracy when the class labels are permuted [36]. This rank was then used for model building by subsequently adding one feature at a time in a “top-down” forward manner (Figure 2). 

### 3.2. Comparison of Classification Methods: Internal Validation Performance

To reduce variability, and complexity and to keep validation parameters as constant as possible, the performance of the classifiers was tested within the same dataset by a ten-times repeated 10-fold cross-validation (Figure 2). This validation scheme partitions the training data into 10 nearly equal-sized folds. Subsequently, 10 iterations of training and validation are performed. During each of these iterations a different fold of the training data is left out for validation and the remaining are used for learning. The mean accuracy of all 10-folds validated is thus the 10-fold cross-validated (10 × 10 CV) accuracy of the model. By repeating this process 10 times a more robust and unbiased estimation of the performance is obtained. In our study, the balanced accuracy (bAcc), defined as the mean of sensitivity and specificity, was used as a performance measure. It should be noted that the individual classification performances are artificially elevated due to information leakages, caused by AM and RO being used for primary feature selection, and that the entire AM and RO datasets are used for importance ranking prior to cross-validation. However, the differences between the individual classification method performances are assumed, unaffected by these leakages.

**Figure 3:**
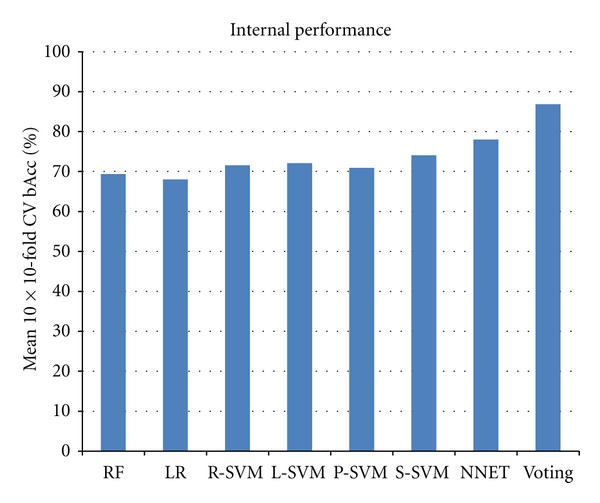
Internal validation performance. Shown in blue histograms are the mean 10-times repeated 10-fold cross-validation balanced accuracy performance (bAcc) within the two training datasets: AM and RO. Methods used are random forest (RF), logistic regression (LR), support vector machines with a radial (R-SVM), linear (L-SVM), polynomial (P-SVM), sigmoid kernel (S-SVM), a neural network with a single hidden layer (NNET), or cross-method voting (Voting).

The performances within the AM and RO by each classification method were combined, and the mean performance calculated. The classifiers based on NNET had the best performance achieving a mean 10 × 10 CV bAcc of 78%, followed by S-SVM, L-SVM, R-SVM, P-SVM, RF, and LR achieving mean 10 × 10 CV bAcc of 74.1%, 72.1%, 71.6%, 70.9%, 69.4%, and 68.0%, respectively (Figure 3 and Supplementary Table 2). The significance of these differences was tested using the down-sampling statistical test described in Section 2, showing that NNET significantly outperformed RF (*P* = 0.011), LR (*P* = 1.2*e* ^−5^), L-SVM (*P* = 0.027), and P-SVM (*P* = 6.3*e* ^−4^). NNET only borderline significantly outperformed R-SVM (*P* = 0.07) and S-SVM (*P* = 0.10). Furthermore, S-SVM also performed significantly better than RF (*P* = 0.049), LR (*P* = 9.0*e* ^−5^), and P-SVM (*P* = 0.049), and R-SVM outperformed RF (*P* = 0.018). No significant performance difference was found when comparing the other classification methods.

We next combined the cross-validated results by the seven methods into a voting procedure. This led to a mean 10 × 10 CV bAcc of 86.9%, which significantly outperformed all the seven underlying classification methods: RF (*P* = 1.1*e* ^−19^), LR (*P* = 1.7*e* ^−18^), R-SVM (*P* = 4.2*e* ^−12^), L-SVM (*P* = 1.4*e* ^−14^), P-SVM (*P* = 8.9*e* ^−16^), S-SVM (*P* = 5.8*e* ^−11^), and NNET (*P* = 2.6*e* ^−4^) (Figure 3 and Supplementary Table 2).

### 3.3. External Validation Performance between Similar Microarray Platforms

**Figure 4:**
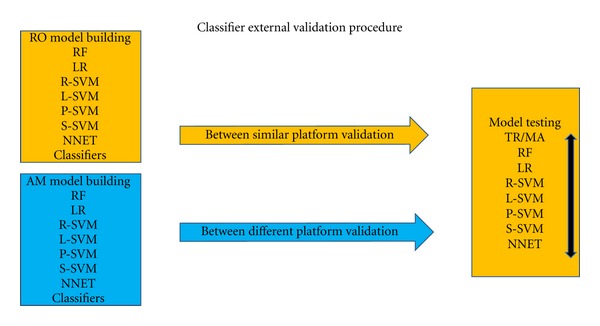
The procedure for external validation of classifiers. External classifier validation. Two datasets were used for training (AM and RO), and two others for testing (TR and MA). Datasets based on the Affymetrix and Agilent platforms are shown in orange and blue, respectively. RO and AM classifiers were used for evaluating external validation of classifiers developed from datasets using similar platform and using different microarray platforms, respectively. The models built in RO were tested in TR and MA and their mean performance calculated. This was done for all classification methods and compared. The same was done for testing AM models.

The performance of the classifiers was validated in independent datasets based on the same microarray platform (Affymetrix), which covers the validation of RO-based classifiers on the TR and MA test data (Figure 4), which contained between 7 to 35 features (Table 2). In this setting, the entire classifiers developed in the training set, using the features and rules associated with the classifiers, were used to classify the independent samples in the entire test sets, and the performance is defined as the mean test accuracy in TR and MA.

**Figure 5:**
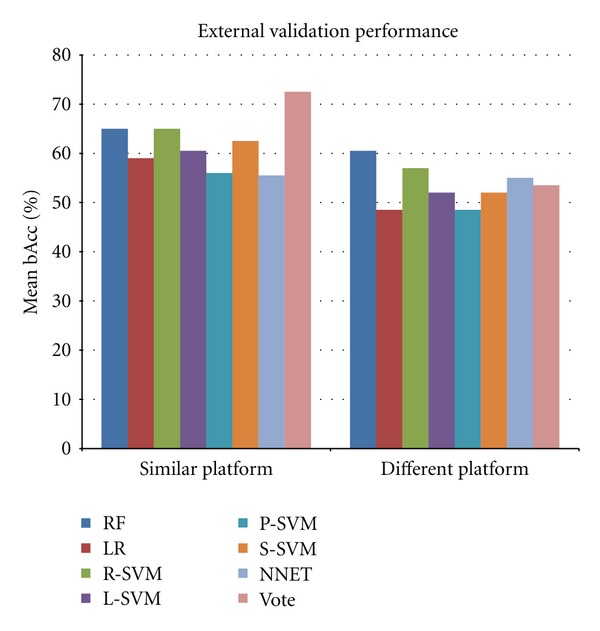
External validation performance. External classifier performance. The histogram shows the mean testing performance when classifiers are validated in test sets based on similar platforms or different platforms as from which they were developed. Each bar represents the mean balanced accuracy by random forest (RF), logistic regression (LR), support vector machines with a radial (R-SVM), linear (L-SVM), polynomial (P-SVM), sigmoid kernel (S-SVM), a neural network with a single hidden layer (NNET), or cross-method voting (VOTE), respectively.

RF and R-SVM had the best external classification performance achieving a mean bAcc of 65%, while NNET had the poorest performance (55.5% mean bAcc) (Figure 5). RF performed significantly better than LR (*P* = 0.0085), L-SVM (*P* = 0.026), P-SVM (*P* = 0.0057), and NNET (*P* = 0.0012), and R-SVM also performed significantly better than LR (*P* = 0.0064), L-SVM (*P* = 0.025), P-SVM (*P* = 0.013) and NNET (*P* = 0.0025) (Figure 5). 

The voting procedure increased the performance to a 72.5% mean bAcc and significantly outperformed the seven underlying methods: RF (*P* = 6.1*e* ^−5^), LR (*P* = 1.5*e* ^−8^), R-SVM (*P* = 0.00026), L-SVM (*P* = 6.0*e* ^−8^), P-SVM (*P* = 2.1*e* ^−7^), S-SVM (*P* = 1.9*e* ^−6^), and NNET (*P* = 4.7*e* ^−7^) (Figure 5). Detailed overview of the individual validation results is shown in Supplementary Table 3.

### 3.4. External Validation Performance between Different Microarray Platforms

The performance of the classifiers was finally validated in independent datasets (Affymetrix) based on a different microarray platform from the one used by training data (Agilent) and covers the validation of AM-based classifiers on the TR and MA test data. These classifiers contained 4 to 21 features (Table 2). As in the case of the between-similar-platform validation, the entire classifier developed in the training set, using the features and rules associated with the classifiers, was used to classify the independent samples in the entire test sets (Figure 4), and the performance is defined as the mean test accuracy in TR and MA.

Comparison of classifiers developed on an Agilent dataset and validated on an Affymetrix dataset revealed that the mean classification performances based on RF had the best performance amongst the seven methods, achieving a mean bAcc of 60.5%, while the poorest performances were achieved by the LR- and P-SVM classifiers, which obtained only 48.5% bAcc (Figure 5). RF performed significantly better than the other six methods: LR (*P* = 3*e* ^−4^), R-SVM (*P* = 0.038), L-SVM (*P* = 0.0012), P-SVM (*P* = 9.8*e* ^−5^), S-SVM (*P* = 0.0015), and NNET (*P* = 0.032) (Figure 5). In contrast to the between-similar-platforms validations, the voting procedure only obtained a mean bAcc performance of 53.5%, which was a borderline significantly inferior to RF (*P* = 0.059) (Figure 5). Detailed description of the individual between different platforms validation results is shown in Supplementary Table 4.

## 4. Conclusion and Discussion

This study compared seven classification methods and a voting procedure ability to predict metastasis outcome in lymph node-negative breast cancer patients. The results showed that during internal assessment and external validation—methods based on voting had the best performance. 

Our study first compared the internal performance within a single dataset and showed that NNET had the best performance followed by the support vector machines, while RF and LR had the worst performances. This implies that at least for prediction of metastasis outcome within the same dataset—NNET and support vector machines displays superiority. This finding agrees well with other studies using cross- or hold-out procedures for performance comparisons. For example, one study comparing the performance of eight different classification methods showed that NNET and SVMs in general perform better than the other six methods for predicting outcome in eight different cancer microarray datasets [15]. Several studies confirm our finding of RF inferiority when using cross-validation [17, 18, 40]. Interestingly, a study conducting algorithm comparison on microarray gene expression based drug signatures showed that NNET and R-SVM had the best performance when tested in the most heterogeneous datasets [41]. As the datasets used in our study are likely to be very heterogeneous, due to the nature and etiology of breast cancer, the superior performances of NNET and support vector machines could reflect the ability of these particular methods to distinguish outcome in such complex datasets. 

Combining the classification results by each method into classification based on voting significantly increased the internal performance. The finding of voting superiority in the internal validations suggests that voting would be valuable when applied to datasets having a combination of limited technical variation (due to using same protocols and platforms) and biological heterogeneity. Although the patients in our study are limited to being node negative, they may still be very heterogeneous due to the existence of various breast cancer molecular subgroups and the disease etiology. Voting may therefore reduce the variation associated with this biological heterogeneity. This is in line with the above-mentioned study, showing that some classification methods are more suitable for prediction tasks in complex datasets [41].

Our finding of voting superiority agrees with four other studies: one using multiple different feature extraction methods in combination with SVM for gene microarray classification showed that using a voting-based method across all the examined combinations achieved a better 10-fold cross-validated classification performance compared to any single combination [33]; a second study showed that an SVM-based ensemble outperformed single SVM for microarray data classification [42]; a third study comparing the performance of principal component discriminant classifiers either with or without voting using cross-validation applied on a simulated dataset a leukemia microarray gene expression dataset, a Gaucher serum proteomics dataset and a grape extract metabolomics dataset, also showed that voting had a better performance than the nonvoting method [43]; a fourth study comparing the performance of single models to combined models in thirteen diverse microarray datasets, which included predictions of estrogen receptor positivity and complete pathological response to chemotherapy in breast cancer, found that the majority of combined multiple models had a better classification performance than the single models [21]. Furthermore, our findings also agree with a study by Taylor and Kim who, by splitting their original datasets into training and test parts, showed that voting based on nearest mean voters was a top performing method with respect to classification on lung or prostate cancer data. In contrast to our results, RF was found to perform equally well as the mean voter [44]. However, this discrepancy is likely caused by the difference in classification tasks. In contrast to our results, Statnikov and coworkers found that SVM-based ensemble/voting methods perform similar or worse compared to SVM nonensemble/voting methods, when tested on ten different human gene expression datasets by 10-fold cross-validation. However, these comparisons were primarily based on multicategory classification [45]. 

In the second experiment, we investigated the classifiers performance when tested in an external dataset based on a similar microarray platform. In this setup, RF and R-SVM achieved the best performances, both significantly outperforming four of the five remaining classification methods. Furthermore, the voting procedure significantly outperformed the seven underlying classification methods. This suggests that every classifier in the voting committee agrees on most of the samples that are predicted correctly and that the majority of voters do not make the same misclassifications. The finding that voting and RF have the best performances could be explained by these methods' ability to reduce the cross-study prediction variance, without simultaneously increasing prediction bias [37]. Only a limited number of studies have compared the cross-study performance of multiple classification methods. A study by Tan and Gilbert compared the performance of single C4.5 classifiers with the voting-like C4.5 bagging and boosting classifiers on gene expression data cancer classification. In four of the experiments, an independent dataset measured on similar platforms was used for testing. Interestingly, one of the results found that bagging and boosting performed better than single C4.5 classifiers when predicting relapse within a 5-year period in a small group of 19 independent breast cancer patients, achieving 88.7%, 88.7%, and 75% bAcc, respectively [46]. These voting results are higher than the mean voting performance achieved in our two test sets (72.5% bAcc). This could be due to three factors: (1) the training and testing sets used by Tan and Gilbert originate from the same population (The Netherlands), and the sample preparations and gene expression measurements were performed using the same protocols; (2) the classification task also differs. It might be easier to predict relapse within a 5-year period than predicting if a patient would ever metastasize; (3) the voting methods used also differ. Another study deployed a committee of neural networks for gene expression based leukemia subclassification using three gene expression datasets measured on the same microarray platform. The study used a first dataset for feature selection, a second for network training and committee development, and a third independent test set for validating the committee. When compared to the performance by each of the underlying classifiers in the committee, the committee neural networks proved to perform better or equally well in the final testing set [47]. 

In the third experiment, the trained classifiers were externally validated on datasets based on a different microarray platform. With this setup, the performances dropped dramatically. This suggests that the data distributions in the Agilent and Affymetrix datasets are dissimilar. This is likely caused by biological and technical variation. The fact that the training and test samples originate from two different patient populations could make the data distributions dissimilar. The technical variation may originate from several sources, for example, the size of the oligonucleotides used, probe coverage, labeling, cross-hybridization, and detection limits by the scanner. Furthermore, the two platforms use different strategies for measuring the same RNA quantity. On the Agilent platform, this quantity is measured as the ratio of fluorescence intensities between a sample and a reference at each spot on the array, while the Affymetrix platform uses single channel measurements for a collection of probe sets covering one gene, which are therefore not comparable. To circumvent this obstacle, we standardized the datasets. However, this standardization seemed not to be sufficient for avoiding a drop in performance by all the classification methods used. Therefore, it is likely that the data distributions of the training and test sets are very heterogeneous thus hampering the external application of the classifiers.

Although all classification methods experience a drop in performance when validating between datasets measured on different platforms, the results showed that RF remained the strongest method and significantly outperformed the six other methods. Surprisingly, the voting procedure performed poorly when validated on data measured on a different microarray platform and was a borderline significantly outperformed by RF. This is probably due to randomness by each method/voter. The finding of RF performing better than the cross-method voting procedure suggests that when tested on datasets using a different microarray technology for gene expression measurement, voting procedures based on the same classification algorithm, in the case of random forest being a collection of decision trees, are more advantageous than voting procedures based on diverse classification methods. This implies that RF compared to voting is more capable of reducing the prediction variance associated with validation across studies and platforms. Therefore, in a situation when validating between different microarray platforms and where voting is outperformed, an approach called bagging might prove advantageous. Bagging uses voters consisting of multiple classifiers developed by bootstrap resamplings from the same dataset and based on the same classification method (decisions trees in the case of random forest) [48]. Thus, bagged SVM, LR, and NNET might be considered ideal for cross-study-cross-platform validations. RF may also be powerful, as the method is based on multiple decision rules, which might be better at segregating a complex data structure. This situation is in line with a study showing that molecular classification of cancer achieves better or similar performance as other classification algorithms, when using decision rules based on a single gene or a gene pair [49].

In the literature, there has been a limited number of studies comparing the performance of multiple classification methods, applied to across the dataset and microarray platform validations. One study by Yoshida compared a nearest template prediction method (NTP) with CART (single decision tree method), weighted voting, SVM, and k-nearest neighbor classification (k-NN) across datasets using Agilent datasets for training and Affymetrix datasets for testing [20]. For prediction of estrogen receptor positivity in breast cancer, NTP had the best performance, while SVM had the worst performance. For predictions of breast cancer molecular subtypes, SVM had the best performance in two of three testing sets used for this purpose, while NTP had the best performance in the third dataset. The worst performing methods were achieved by CART and k-NN [20]. In our study, SVM was not a top performing method for cross-platform testing. These differences are likely due to two factors: first, the study by Hoshida did not apply the entire classifier to the test sets, but only the list of genes defined by the training datasets. This list was used to train and test a classifier in the validation dataset; second, the study addresses completely different classification tasks compared to our study.

Our results showed that when validation is applied between two datasets of similar or different microarray platforms, LR and NNET were among the poorest performing methods. 

The general poor performance of LR could be due to several factors. First, a strong LR model is frequently composed of predictors being highly univariate significant and remains significant in the multivariate model. The fact that the list of 519 rank-significant genes defined by the eight feature definer datasets was reduced to a pool of 283 genes could have led to the exclusion of some highly significant genes, due to the only reason that they were not shared by the AM, RO, TR, and MA datasets, thus impairing the possibility for development of a stronger model. This could explain the poor performance by LR classifiers developed by the individual AM and RO datasets; second, an LR model requires a large sample size for providing robust maximum likelihood parameter estimation. Although the training datasets contain 151 and 286 samples, these sample sizes may not be sufficient for developing a strong model if some of the highly discriminative genes are absent; third, LR models rely on the assumption that there is no colinearity between the variables, meaning that the variables/features should be independent from each other. This assumption may be violated if predictors in a logistic model consist of, for example, gene expression features, some of which could be coregulated, thus leading to colinearity and weakening the model; finally, LR is sensitive to outliers. As we have not removed any samples from our datasets, and the possibility of outlier presence thus could be evident, this could also hamper the predictive power of the LR models.

The finding of NNET had a high internal 10-fold cross validation performance but a weak external validation performance could suggest that the NNET classifiers are not very generable. Another explanation could be that the transfer function used by the neural network was a sigmoid function, which is identical to that used in logistic regression, thus leading to some of the weaknesses observed in logistic regression, although the parameter estimation in neural networks is not conducted by maximum-likelihood but by a gradient descent algorithm. Interestingly, a study has compared the classification performance of four different single hidden layer feedforward neural networks on three microarray gene expression cancer dataset, showing that an SVD-neural classifier based on a *tansig* activation function and using single value decomposition for parameter estimation had a better performance compared to the three other methods and that this classifier outperformed support vector machines, principle component analysis classifiers, and Fisher discriminant analysis classifiers [50]. This implies that using another neural network type could achieve a better performance when applied for external validation in datasets based on similar or different microarray platforms.

In conclusion, voting-based classifiers provided an advantage with respect to classifying metastasis outcome in breast cancer patients. When testing was performed within the same dataset or between datasets using similar microarray platforms, combining class decisions by multiple classification methods significantly increased the classification performance. Random forest, a voting-like method, proved to be the strongest method when testing was performed in datasets based on a different microarray platform.

## Supplementary Material

Supplementary Table 1 shows list of 283 rank-significant genes for classifier building, by gene symbol (left column) and by description (right column).
Supplementary Table 2 shows the internal validation results within the AM and RO datasets and the mean of AM & RO. These results are shown with respect to sensitivity (Sen), specificity (Spe) and balanced accuracy (bAcc) based on the ten times repeated ten folds cross-validation by each of the classification methods used. The classification are as follows: random forest (RF), logistic regression (LR), support vector machines with a radial (R-SVM), linear (L-SVM), polynomial (P-SVM), sigmoid kernel (S-SVM), a neural network with a single hidden layer (NNET) or cross method voting (Voting).
Supplementary Table 3 shows the testing results by classifiers developed in the RO dataset and validated in TR or MA and the combined mean performance in TR and MA. These results are shown with respect to sensitivity (Sen), specificity (Spe) and balanced accuracy (bAcc), by each of the classification methods used. The classification are as follows: random forest (RF), logistic regression (LR), support vector machines with a radial (R-SVM), linear (L-SVM), polynomial (P-SVM), sigmoid kernel (S-SVM), a neural network with a single hidden layer (NNET) or cross method voting (Voting).
Supplementary Table 4 shows the testing results by classifiers developed in the AM dataset and validated in TR or MA and the combined mean performance in TR and MA. These results are shown with respect to sensitivity (Sen), specificity (Spe) and balanced accuracy (bAcc), by each of the classification methods used. The classification are as follows: random forest (RF), logistic regression (LR), support vector machines with a radial (R-SVM), linear (L-SVM), polynomial (P-SVM), sigmoid kernel (S-SVM), a neural network with a single hidden layer (NNET) or cross method voting (Voting).

## Figures and Tables

**Table 1:** Overview of datasets used.

Dataset	Chip	Probes (*K*)	Patients	Outcome	Treatment	Define genes	Internal CV	External validation train	External validation test	Reference
Amsterdam	Agilent/Rosetta	25	295, N^+^, N^−^	DM	None, et, ct	*√*				[31]
Amsterdam (AM) (subset of the above)	Agilent/Rosetta	25	151, N^−^	DM	None	*√*	*√*	*√*		[31]
Rotterdam (RO)	Affymetrix HG-133A	22	286, N^−^	DM	None	*√*	*√*	*√*		[28]
HUMAC	Spotted oligonucleotides	29	60, N^−^	ME	None	*√*				[4]
Huang	Affymetrix 95av2	12	52, N^+^	RE	ct	*√*				[27]
Sotiriou 2003	Spotted cDNA	7.6	99, N^+^/N^−^	RE	et, ct	*√*				[24]
Sotiriou 2006	Affymetrix HG-133A	22	179, N^+^/N^−^	DM	et	*√*				[25]
Uppsala	Affymetrix HG-133A+B	44	236, N^+^/N^−^	DF	None, ct, et	*√*				[6]
Stockholm	Affymetrix HG-133A+B	44	159, N^+^/N^−^	RE	None, ct, et	*√*				[23]
TRANSBIG (TR)	Affymetrix HG-133A	22	147, N^−^	DM	None				*√*	[32]
Mainz (MA)	Affymetrix HG-133A	22	200, N^−^	DM	None				*√*	[33]

The columns show the following: “dataset”: the individual names for the eight datasets; “chip”: microarray chip used; “probes”: number of probes on the chip measured in thousands (*K* = 1000); patients': number of patients in the study and their nodal status (N^+^ and N^−^ indicates number of node-positive and -negative patients; “outcome” covers the clinical outcome being DM: distant metastasis, ME: metastasis, RE: relapse, and DF: death from breast cancer; “treatment” shows patient treatments abbreviated by et: endocrine therapy, ct: chemo therapy, and none: no adjuvant therapy.

**Table 2:** Number of features in the models.

Dataset	AM	RO
Method	(*n* = 151)	(*n* = 286)

RF	21	21
LR	5	11
R-SVM	20	25
L-SVM	8	11
P-SVM	4	7
S-SVM	17	35
NNET	21	16

AM and RO are the Amsterdam and Rotterdam training sets and *n* shows the number of samples in the respective datasets. Methods used are as follows: RF: random forest, LR: logistic regression, R-, L-, P-, and S-SVM: support vector machine with a radial basis function, linear, polynomial, or sigmoid kernel, and NNET: neural network with a single hidden layer.
