# Supplementary material for: Gene Expression Profiles for Predicting Metastasis in Breast Cancer: A Cross-Study Comparison of Classification Methods
Source: ScientificWorldJournal. 2012 Nov 28;2012:380495. doi: 10.1100/2012/380495 (PMC3515909; doi:10.1100/2012/380495)
Supplement: Supplementary file 1 — Supplementary Table 1 shows list of 283 rank-significant genes for classifier building, by gene symbol (left column) and by description (right column). Supplementary Table 2 shows the internal validation results within the AM and RO datasets and the mean of AM & RO. These results are shown with respect to sensitivity (Sen), specificity (Spe) and balanced accuracy (bAcc) based on the ten times repeated ten folds cross-validation by each of the classification methods used. The classification are as follows: random forest (RF), logistic regression (LR), support vector machines with a radial (R-SVM), linear (L-SVM), polynomial (P-SVM), sigmoid kernel (S-SVM), a neural network with a single hidden layer (NNET) or cross method voting (Voting). Supplementary Table 3 shows the testing results by classifiers developed in the RO dataset and validated in TR or MA and the combined mean performance in TR and MA. These results are shown with respect to sensitivity (Sen), specificity (Spe) and balanced accuracy (bAcc), by each of the classification methods used. The classification are as follows: random forest (RF), logistic regression (LR), support vector machines with a radial (R-SVM), linear (L-SVM), polynomial (P-SVM), sigmoid kernel (S-SVM), a neural network with a single hidden layer (NNET) or cross method voting (Voting). Supplementary Table 4 shows the testing results by classifiers developed in the AM dataset and validated in TR or MA and the combined mean performance in TR and MA. These results are shown with respect to sensitivity (Sen), specificity (Spe) and balanced accuracy (bAcc), by each of the classification methods used. The classification are as follows: random forest (RF), logistic regression (LR), support vector machines with a radial (R-SVM), linear (L-SVM), polynomial (P-SVM), sigmoid kernel (S-SVM), a neural network with a single hidden layer (NNET) or cross method voting (Voting). [file 380495.f1.docx]

**Supplementary Table 1. The list of 283 rank significant genes.**

| **Gene symbol** | **Descriptionolonne2** |
| --- | --- |
| ABCA5 | ATP-binding cassette, sub-family A (ABC1), member 5 |
| ABCA8 | ATP-binding cassette, sub-family A (ABC1), member 8 |
| ABCC10 | ATP-binding cassette, sub-family C (CFTR/MRP), member 10 |
| ABCC5 | ATP-binding cassette, sub-family C (CFTR/MRP), member 5 |
| ABTB2 | ankyrin repeat and BTB (POZ) domain containing 2 |
| ACD | adrenocortical dysplasia homolog (mouse) |
| ADFP | adipose differentiation-related protein |
| ADH1B | alcohol dehydrogenase IB (class I), beta polypeptide |
| ADRA2A | adrenergic, alpha-2A-, receptor |
| ADRM1 | adhesion regulating molecule 1 |
| ALDH1A1 | aldehyde dehydrogenase 1 family, member A1 |
| ALDH2 | aldehyde dehydrogenase 2 family (mitochondrial) |
| ALDH6A1 | aldehyde dehydrogenase 6 family, member A1 |
| APOD | apolipoprotein D |
| ARHGEF6 | Rac/Cdc42 guanine nucleotide exchange factor (GEF) 6 |
| ATP1B3 | ATPase, Na+/K+ transporting, beta 3 polypeptide |
| ATP2A2 | ATPase, Ca++ transporting, cardiac muscle, slow twitch 2 |
| ATP9A | ATPase, Class II, type 9A |
| AURKB | aurora kinase B |
| BARD1 | BRCA1 associated RING domain 1 |
| BCL2 | B-cell CLL/lymphoma 2 |
| BCL2L1 | BCL2-like 1 |
| BRCA1 | breast cancer 1, early onset |
| BUB1 | BUB1 budding uninhibited by benzimidazoles 1 homolog (yeast) |
| BUB1B | BUB1 budding uninhibited by benzimidazoles 1 homolog beta (yeast) |
| C6 | complement component 6 |
| C7ORF24 | chromosome 7 open reading frame 24 |
| CACNA1D | calcium channel, voltage-dependent, L type, alpha 1D subunit |
| CARS | cysteinyl-tRNA synthetase |
| CAT | catalase |
| CCNA2 | cyclin A2 |
| CCNB1 | cyclin B1 |
| CCNB2 | cyclin B2 |
| CCNE2 | cyclin E2 |
| CCNF | cyclin F |
| CCT5 | chaperonin containing TCP1, subunit 5 (epsilon) |
| CCT6A | chaperonin containing TCP1, subunit 6A (zeta 1) |
| CD44 | CD44 molecule (Indian blood group) |
| CDC2 | cell division cycle 2, G1 to S and G2 to M |
| CDC20 | CDC20 cell division cycle 20 homolog (S. cerevisiae) |
| CDC25B | cell division cycle 25B |
| CDC25C | cell division cycle 25C |
| CDC34 | cell division cycle 34 |
| CDC45L | CDC45 cell division cycle 45-like (S. cerevisiae) |
| CDK8 | cyclin-dependent kinase 8 |
| CDKN3 | cyclin-dependent kinase inhibitor 3 (CDK2-associated dual specificity phosphatase) |
| CDO1 | cysteine dioxygenase, type I |
| CENPE | centromere protein E, 312kDa |
| CENPF | centromere protein F, 350/400ka (mitosin) |
| CH25H | cholesterol 25-hydroxylase |
| CHAF1B | chromatin assembly factor 1, subunit B (p60) |
| CIRBP | cold inducible RNA binding protein |
| CKAP5 | cytoskeleton associated protein 5 |
| CKS2 | CDC28 protein kinase regulatory subunit 2 |
| CNN3 | calponin 3, acidic |
| CNTN1 | contactin 1 |
| CP | ceruloplasmin (ferroxidase) |
| CREBL2 | cAMP responsive element binding protein-like 2 |
| CRIM1 | cysteine rich transmembrane BMP regulator 1 (chordin-like) |
| CSE1L | CSE1 chromosome segregation 1-like (yeast) |
| CSTF1 | cleavage stimulation factor, 3' pre-RNA, subunit 1, 50kDa |
| CTPS | CTP synthase |
| CTSD | cathepsin D (lysosomal aspartyl peptidase) |
| CTSL | cathepsin L |
| CX3CR1 | chemokine (C-X3-C motif) receptor 1 |
| CYP4B1 | cytochrome P450, family 4, subfamily B, polypeptide 1 |
| CYP4F12 | cytochrome P450, family 4, subfamily F, polypeptide 12 |
| DDIT4 | DNA-damage-inducible transcript 4 |
| DDX39 | DEAD (Asp-Glu-Ala-Asp) box polypeptide 39 |
| DLG7 | discs, large homolog 7 (Drosophila) |
| DLX2 | distal-less homeobox 2 |
| DOCK1 | dedicator of cytokinesis 1 |
| DPT | dermatopontin |
| DUSP1 | dual specificity phosphatase 1 |
| DUSP4 | dual specificity phosphatase 4 |
| DYRK2 | dual-specificity tyrosine-(Y)-phosphorylation regulated kinase 2 |
| EBP | emopamil binding protein (sterol isomerase) |
| EDG1 | endothelial differentiation, sphingolipid G-protein-coupled receptor, 1 |
| EGR2 | early growth response 2 (Krox-20 homolog, Drosophila) |
| ELOVL5 | ELOVL family member 5, elongation of long chain fatty acids (FEN1/Elo2, SUR4/Elo3-like, yeast) |
| ENPP2 | ectonucleotide pyrophosphatase/phosphodiesterase 2 (autotaxin) |
| EPHX2 | epoxide hydrolase 2, cytoplasmic |
| ESPL1 | extra spindle poles like 1 (S. cerevisiae) |
| EVPL | envoplakin |
| EXO1 | exonuclease 1 |
| EZH2 | enhancer of zeste homolog 2 (Drosophila) |
| F3 | coagulation factor III (thromboplastin, tissue factor) |
| FADD | Fas (TNFRSF6)-associated via death domain |
| FANCG | Fanconi anemia, complementation group G |
| FAS | Fas (TNF receptor superfamily, member 6) |
| FBLN1 | fibulin 1 |
| FBLN5 | fibulin 5 |
| FCER1A | Fc fragment of IgE, high affinity I, receptor for; alpha polypeptide |
| FEN1 | flap structure-specific endonuclease 1 |
| FGL2 | fibrinogen-like 2 |
| FLJ22531 | - |
| FMO2 | flavin containing monooxygenase 2 (non-functional) |
| FOS | v-fos FBJ murine osteosarcoma viral oncogene homolog |
| FOXM1 | forkhead box M1 |
| FRZB | frizzled-related protein |
| FUCA1 | fucosidase, alpha-L- 1, tissue |
| GABARAP | GABA(A) receptor-associated protein |
| GAD1 | glutamate decarboxylase 1 (brain, 67kDa) |
| GALK1 | galactokinase 1 |
| GEM | GTP binding protein overexpressed in skeletal muscle |
| GGCX | gamma-glutamyl carboxylase |
| GLA | galactosidase, alpha |
| GLI1 | glioma-associated oncogene homolog 1 (zinc finger protein) |
| GMPS | guanine monphosphate synthetase |
| GNG11 | guanine nucleotide binding protein (G protein), gamma 11 |
| GNG12 | guanine nucleotide binding protein (G protein), gamma 12 |
| GPSM2 | G-protein signalling modulator 2 (AGS3-like, C. elegans) |
| GRIK1 | glutamate receptor, ionotropic, kainate 1 |
| GSTM3 | glutathione S-transferase M3 (brain) |
| GUK1 | guanylate kinase 1 |
| GYS2 | glycogen synthase 2 (liver) |
| H2AFZ | H2A histone family, member Z |
| HIST1H3D | histone cluster 1, H3d |
| HMGB2 | high-mobility group box 2 |
| HMMR | hyaluronan-mediated motility receptor (RHAMM) |
| HNMT | histamine N-methyltransferase |
| HNRPAB | heterogeneous nuclear ribonucleoprotein A/B |
| HNRPH2 | heterogeneous nuclear ribonucleoprotein H2 (H') |
| HPN | hepsin (transmembrane protease, serine 1) |
| HPRT1 | hypoxanthine phosphoribosyltransferase 1 (Lesch-Nyhan syndrome) |
| IFNGR2 | interferon gamma receptor 2 (interferon gamma transducer 1) |
| IGFBP4 | insulin-like growth factor binding protein 4 |
| IQGAP2 | IQ motif containing GTPase activating protein 2 |
| ITM2A | integral membrane protein 2A |
| ITPR1 | inositol 1,4,5-triphosphate receptor, type 1 |
| JAK2 | Janus kinase 2 (a protein tyrosine kinase) |
| KCTD12 | potassium channel tetramerisation domain containing 12 |
| KIF11 | kinesin family member 11 |
| KIF13B | kinesin family member 13B |
| KIF14 | kinesin family member 14 |
| KIF2C | kinesin family member 2C |
| KIFC1 | kinesin family member C1 |
| KIAA0101 | KIAA0101 |
| KIAA0247 | KIAA0247 |
| KIAA0286 | - |
| KIAA0319 | KIAA0319 |
| LAMA2 | laminin, alpha 2 (merosin, congenital muscular dystrophy) |
| LARP1 | La ribonucleoprotein domain family, member 1 |
| LEP | leptin (obesity homolog, mouse) |
| LIG1 | ligase I, DNA, ATP-dependent |
| LMNB1 | lamin B1 |
| LMO2 | LIM domain only 2 (rhombotin-like 1) |
| LPHN2 | latrophilin 2 |
| LPL | lipoprotein lipase |
| LRIG1 | leucine-rich repeats and immunoglobulin-like domains 1 |
| LRP2 | low density lipoprotein-related protein 2 |
| MAD2L1 | MAD2 mitotic arrest deficient-like 1 (yeast) |
| MAPRE1 | microtubule-associated protein, RP/EB family, member 1 |
| MARS | methionine-tRNA synthetase |
| MCM3 | MCM3 minichromosome maintenance deficient 3 (S. cerevisiae) |
| MCM6 | MCM6 minichromosome maintenance deficient 6 (MIS5 homolog, S. pombe) (S. cerevisiae) |
| MCM7 | MCM7 minichromosome maintenance deficient 7 (S. cerevisiae) |
| MEIS1 | Meis1, myeloid ecotropic viral integration site 1 homolog (mouse) |
| MELK | maternal embryonic leucine zipper kinase |
| MGP | matrix Gla protein |
| MKI67 | antigen identified by monoclonal antibody Ki-67 |
| MN1 | meningioma (disrupted in balanced translocation) 1 |
| MRPL12 | mitochondrial ribosomal protein L12 |
| MT2A | metallothionein 2A |
| MTHFD2 | methylenetetrahydrofolate dehydrogenase (NADP+ dependent) 2, methenyltetrahydrofolate cyclohydrolase |
| MVD | mevalonate (diphospho) decarboxylase |
| MYBL2 | v-myb myeloblastosis viral oncogene homolog (avian)-like 2 |
| NCOA1 | nuclear receptor coactivator 1 |
| NDUFA9 | NADH dehydrogenase (ubiquinone) 1 alpha subcomplex, 9, 39kDa |
| NEDD9 | neural precursor cell expressed, developmentally down-regulated 9 |
| NEK2 | NIMA (never in mitosis gene a)-related kinase 2 |
| NME5 | non-metastatic cells 5, protein expressed in (nucleoside-diphosphate kinase) |
| NNAT | neuronatin |
| NP | nucleoside phosphorylase |
| NR3C2 | nuclear receptor subfamily 3, group C, member 2 |
| NTRK2 | neurotrophic tyrosine kinase, receptor, type 2 |
| NUDT1 | nudix (nucleoside diphosphate linked moiety X)-type motif 1 |
| NUP155 | nucleoporin 155kDa |
| NUP62 | nucleoporin 62kDa |
| NVL | nuclear VCP-like |
| OMD | osteomodulin |
| P4HA2 | procollagen-proline, 2-oxoglutarate 4-dioxygenase (proline 4-hydroxylase), alpha polypeptide II |
| PDCD4 | programmed cell death 4 (neoplastic transformation inhibitor) |
| PDE4A | phosphodiesterase 4A, cAMP-specific (phosphodiesterase E2 dunce homolog, Drosophila) |
| PDZRN3 | PDZ domain containing RING finger 3 |
| PFKP | phosphofructokinase, platelet |
| PHLDA2 | pleckstrin homology-like domain, family A, member 2 |
| PIN1 | protein (peptidylprolyl cis/trans isomerase) NIMA-interacting 1 |
| PIP | prolactin-induced protein |
| PIR | pirin (iron-binding nuclear protein) |
| PKMYT1 | protein kinase, membrane associated tyrosine/threonine 1 |
| PLK4 | polo-like kinase 4 (Drosophila) |
| PLP2 | proteolipid protein 2 (colonic epithelium-enriched) |
| PNMA2 | paraneoplastic antigen MA2 |
| PNRC1 | proline-rich nuclear receptor coactivator 1 |
| POLD1 | polymerase (DNA directed), delta 1, catalytic subunit 125kDa |
| POLR2H | polymerase (RNA) II (DNA directed) polypeptide H |
| POLS | polymerase (DNA directed) sigma |
| PRAME | preferentially expressed antigen in melanoma |
| PSD3 | pleckstrin and Sec7 domain containing 3 |
| PSMB3 | proteasome (prosome, macropain) subunit, beta type, 3 |
| PSMB7 | proteasome (prosome, macropain) subunit, beta type, 7 |
| PSMD1 | proteasome (prosome, macropain) 26S subunit, non-ATPase, 1 |
| PSMD11 | proteasome (prosome, macropain) 26S subunit, non-ATPase, 11 |
| PTDSR | phosphatidylserine receptor |
| PTGER3 | prostaglandin E receptor 3 (subtype EP3) |
| PTGER4 | prostaglandin E receptor 4 (subtype EP4) |
| PTPRT | protein tyrosine phosphatase, receptor type, T |
| PTTG1 | pituitary tumor-transforming 1 |
| QDPR | quinoid dihydropteridine reductase |
| RABGGTA | Rab geranylgeranyltransferase, alpha subunit |
| RABIF | RAB interacting factor |
| RAD51 | RAD51 homolog (RecA homolog, E. coli) (S. cerevisiae) |
| RAD51AP1 | RAD51 associated protein 1 |
| RAE1 | RAE1 RNA export 1 homolog (S. pombe) |
| RALA | v-ral simian leukemia viral oncogene homolog A (ras related) |
| RBMS3 | RNA binding motif, single stranded interacting protein |
| RDBP | RD RNA binding protein |
| RECQL4 | RecQ protein-like 4 |
| RFC3 | replication factor C (activator 1) 3, 38kDa |
| RFC4 | replication factor C (activator 1) 4, 37kDa |
| RGS5 | regulator of G-protein signalling 5 |
| RICS | - |
| RNASEH2A | ribonuclease H2, subunit A |
| RRM1 | ribonucleotide reductase M1 polypeptide |
| RRM2 | ribonucleotide reductase M2 polypeptide |
| RTN1 | reticulon 1 |
| SAC3D1 | SAC3 domain containing 1 |
| SC5DL | sterol-C5-desaturase (ERG3 delta-5-desaturase homolog, fungal)-like |
| SDS | serine dehydratase |
| SEC14L2 | SEC14-like 2 (S. cerevisiae) |
| SEC61G | Sec61 gamma subunit |
| SELE | selectin E (endothelial adhesion molecule 1) |
| SEMA3E | sema domain, immunoglobulin domain (Ig), short basic domain, secreted, (semaphorin) 3E |
| SERPINA1 | serpin peptidase inhibitor, clade A (alpha-1 antiproteinase, antitrypsin), member 1 |
| SF3B4 | splicing factor 3b, subunit 4, 49kDa |
| SFRP4 | secreted frizzled-related protein 4 |
| SFRS5 | splicing factor, arginine/serine-rich 5 |
| SH3BGRL | SH3 domain binding glutamic acid-rich protein like |
| SIAHBP1 | - |
| SIX1 | sine oculis homeobox homolog 1 (Drosophila) |
| SLBP | stem-loop (histone) binding protein |
| SLC14A1 | solute carrier family 14 (urea transporter), member 1 (Kidd blood group) |
| SLC16A3 | solute carrier family 16, member 3 (monocarboxylic acid transporter 4) |
| SLC25A1 | solute carrier family 25 (mitochondrial carrier; citrate transporter), member 1 |
| SLC4A7 | solute carrier family 4, sodium bicarbonate cotransporter, member 7 |
| SLIT2 | slit homolog 2 (Drosophila) |
| SMARCA2 | SWI/SNF related, matrix associated, actin dependent regulator of chromatin, subfamily a, member 2 |
| SORBS2 | sorbin and SH3 domain containing 2 |
| SORL1 | sortilin-related receptor, L(DLR class) A repeats-containing |
| SPAG5 | sperm associated antigen 5 |
| SPRY2 | sprouty homolog 2 (Drosophila) |
| SSPN | sarcospan (Kras oncogene-associated gene) |
| SSRP1 | structure specific recognition protein 1 |
| STC2 | stanniocalcin 2 |
| STMN1 | stathmin 1/oncoprotein 18 |
| SURF2 | surfeit 2 |
| TACSTD1 | tumor-associated calcium signal transducer 1 |
| TAT | tyrosine aminotransferase |
| TBCD | tubulin-specific chaperone d |
| TGFB3 | transforming growth factor, beta 3 |
| TIMELESS | timeless homolog (Drosophila) |
| TIMM17B | translocase of inner mitochondrial membrane 17 homolog B (yeast) |
| TLR3 | toll-like receptor 3 |
| TOP2A | topoisomerase (DNA) II alpha 170kDa |
| TPX2 | TPX2, microtubule-associated, homolog (Xenopus laevis) |
| TRIP13 | thyroid hormone receptor interactor 13 |
| TROAP | trophinin associated protein (tastin) |
| TUBA1 | tubulin, alpha 1 |
| TXN | thioredoxin |
| TXNIP | thioredoxin interacting protein |
| TXNRD1 | thioredoxin reductase 1 |
| TYRP1 | tyrosinase-related protein 1 |
| UBE2C | ubiquitin-conjugating enzyme E2C |
| UBE2V2 | ubiquitin-conjugating enzyme E2 variant 2 |
| WDHD1 | WD repeat and HMG-box DNA binding protein 1 |
| WFDC2 | WAP four-disulfide core domain 2 |
| WWP2 | WW domain containing E3 ubiquitin protein ligase 2 |
| XPOT | exportin, tRNA (nuclear export receptor for tRNAs) |
| YWHAZ | tyrosine 3-monooxygenase/tryptophan 5-monooxygenase activation protein, zeta polypeptide |
| ZNF238 | zinc finger protein 238 |
| ZWINT | ZW10 interactor |
| AASS | aminoadipate-semialdehyde synthase |

**Legend supplementary table 1:**

The table shows list of 283 rank-significant genes for classifier building, by gene symbol (left column) and by description (right column).

**Supplementary Table 2: Internal validation results**

| Training set | AM | | | RO | | | Mean AM & RO | | |
| --- | --- | --- | --- | --- | --- | --- | --- | --- | --- |
| Method | Sen | Spe | bAcc | Sen | Spe | bAcc | Sen | Spe | bAcc |
| RF | 77 | 64 | 70 | 94 | 43 | 68 | 85 | 53 | 69 |
| LR | 48 | 91 | 70 | 68 | 64 | 66 | 58 | 78 | 68 |
| R-SVM | 71 | 71 | 71 | 98 | 47 | 72 | 85 | 59 | 72 |
| L-SVM | 77 | 73 | 75 | 78 | 61 | 70 | 77 | 67 | 72 |
| P-SVM | 65 | 83 | 74 | 69 | 66 | 68 | 67 | 75 | 71 |
| S-SVM | 56 | 98 | 77 | 82 | 60 | 71 | 69 | 79 | 74 |
| NNET | 66 | 99 | 83 | 59 | 88 | 73 | 62 | 94 | 78 |
| Voting | 82 | 99 | 90 | 96 | 70 | 83 | 89 | 85 | 87 |

Shown are the internal validation results within the AM and RO datasets and the mean of AM &

Legend supplementary table 2:

Shown are the internal validation results within the AM and RO datasets and the mean of AM & RO. These results are shown with respect to sensitivity (Sen), specificity (Spe) and balanced accuracy (bAcc) based on the ten times repeated ten folds cross-validation by each of the classification methods used. The classification are as follows: random forest (RF), logistic regression (LR), support vector machines with a radial (R-SVM), linear (L-SVM), polynomial (P-SVM), sigmoid kernel (S-SVM), a neural network with a single hidden layer (NNET) or cross method voting (Voting).

**Supplementary Table 3: Between similar platform validations**

| Training set  RO | Test set  TR | | | Test set  MA | | | Mean TR & MA | | |
| --- | --- | --- | --- | --- | --- | --- | --- | --- | --- |
| Method | Sen | Spe | bAcc | Sen | Spe | bAcc | Sen | Spe | bAcc |
| RF | 79 | 49 | 64 | 63 | 69 | 66 | 71 | 59 | 65 |
| LR | 65 | 48 | 57 | 72 | 51 | 61 | 69 | 50 | 59 |
| R-SVM | 81 | 48 | 65 | 67 | 63 | 65 | 74 | 56 | 65 |
| L-SVM | 56 | 58 | 57 | 65 | 62 | 64 | 61 | 60 | 61 |
| P-SVM | 42 | 73 | 57 | 41 | 69 | 55 | 42 | 71 | 56 |
| S-SVM | 65 | 62 | 63 | 63 | 61 | 62 | 64 | 62 | 63 |
| NNET | 46 | 63 | 54 | 48 | 66 | 57 | 47 | 65 | 56 |
| Voting | 79 | 65 | 72 | 72 | 75 | 73 | 76 | 70 | 73 |

Legend to Supplementary Table 3:

Shown are the testing results by classifiers developed in the RO dataset and validated in TR or MA and the combined mean performance in TR and MA. These results are shown with respect to sensitivity (Sen), specificity (Spe) and balanced accuracy (bAcc), by each of the classification methods used. The classification are as follows: random forest (RF), logistic regression (LR), support vector machines with a radial (R-SVM), linear (L-SVM), polynomial (P-SVM), sigmoid kernel (S-SVM), a neural network with a single hidden layer (NNET) or cross method voting (Voting).

**Supplementary Table 4: Between different platform validations**

| Training set  AM | Test set  TR | | | Test set  MA | | | Mean TR & MA | | |
| --- | --- | --- | --- | --- | --- | --- | --- | --- | --- |
| Method | Sen | Spe | bAcc | Sen | Spe | bAcc | Sen | Spe | bAcc |
| RF | 58 | 57 | 57 | 52 | 76 | 64 | 55 | 67 | 61 |
| LR | 19 | 72 | 45 | 22 | 83 | 52 | 21 | 78 | 50 |
| R-SVM | 48 | 62 | 55 | 50 | 69 | 59 | 49 | 66 | 58 |
| L-SVM | 29 | 67 | 48 | 37 | 75 | 56 | 33 | 71 | 52 |
| P-SVM | 21 | 66 | 43 | 30 | 78 | 54 | 26 | 72 | 49 |
| S-SVM | 40 | 67 | 53 | 35 | 68 | 51 | 38 | 68 | 51 |
| NNET | 33 | 72 | 53 | 41 | 75 | 58 | 37 | 74 | 56 |
| Voting | 20 | 80 | 50 | 23 | 92 | 57 | 22 | 86 | 54 |

‘

Legend to Supplementary Table 4:

Shown are the testing results by classifiers developed in the AM dataset and validated in TR or MA and the combined mean performance in TR and MA. These results are shown with respect to sensitivity (Sen), specificity (Spe) and balanced accuracy (bAcc), by each of the classification methods used. The classification are as follows: random forest (RF), logistic regression (LR), support vector machines with a radial (R-SVM), linear (L-SVM), polynomial (P-SVM), sigmoid kernel (S-SVM), a neural network with a single hidden layer (NNET) or cross method voting (Voting).
